# Association between high-altitude polycythemia and hypertension: a cross-sectional study in adults at Tibetan ultrahigh altitudes

**DOI:** 10.1038/s41371-024-00916-3

**Published:** 2024-05-27

**Authors:** Rong Yin, Yunhong Wu, Man Li, Chunrong Liu, Xue Pu, Wen Yi

**Affiliations:** 1 Department of Nephrology, Hospital of Chengdu Office of People’s Government of Tibet Autonomous Region, Chengdu, Sichuan China; 2 Department of Endocrinology, Hospital of Chengdu Office of People’s Government of Tibet Autonomous Region, Chengdu, Sichuan China; 3 Department of Radiology, Hospital of Chengdu Office of People’s Government of Tibet Autonomous Region, Chengdu, Sichuan China; 4 Intensive Care Unit, Hospital of Chengdu Office of People’s Government of Tibet Autonomous Region, Chengdu, Sichuan China; 5 Department of Gastroenterology, Hospital of Chengdu Office of People’s Government of Tibet Autonomous Region, Chengdu, Sichuan China; 6 Surgical Unit, Hospital of Chengdu Office of People’s Government of Tibet Autonomous Region, Chengdu, Sichuan China

**Keywords:** Hypertension, Risk factors

## Abstract

The study aimed to evaluate the association between high-altitude polycythemia and hypertension in adults residing on Anduo County’s plateau, which is located 4700 meters above sea level. A total of 387 individuals participated in the cross-sectional survey conducted between April and May of 2021. Interviews, physical inspections, and laboratory tests were employed to gather information about all of the subjects. The association between high-altitude polycythemia and hypertension was assessed using multivariable logistic regression models. The average age of the 387 participants was 32.6 ± 6.3 years. Of these participants, 260 (67%) were male. The overall prevalence of hypertension was 27.1% (57/380). When stratified by gender, the prevalence was 12.6% (16/127) in females and 34.2% (89/260) in males. The overall prevalence of high-altitude polycythemia was 19.6% (76/387). When stratified by gender, the prevalence was 26.2% (68/260) in males and 6.3% (8/127) in females. During logistic regression analysis, we found that participants with elevated hemoglobin per 10 g/L had a 26% greater risk of hypertension (adjusting for odds ratio [OR], 1.26; 95% confidence interval [CI], 1.11–1.44). Additionally, high-altitude polycythemia greatly increased the risk of hypertension in comparison to non-high-altitude polycythemia (OR, 3.01; 95% CI, 1.66–5.44, *P* < 0.001). The consistency of the results was further demonstrated by stratified and interaction analyses, showing that Hans individuals had a higher risk of hypertension. High-altitude polycythemia is positively associated with hypertension in adults residing at Tibetan ultrahigh altitudes. The results of the investigation may aid in the planning of future research and guide the development of targeted healthcare practices for high-altitude populations, particularly among Han Chinese residents of the Tibetan Plateau.

## Introduction

Hypertension, one of the most prevalent chronic illnesses, significantly increases the risk of both mortality and cardiovascular disease (CVD) [1]. According to estimates, 1.39 billion people (31.1%) worldwide suffered from hypertension in 2010 [2]. The general population in China exhibits a prevalence of hypertension ranging from 18.0% to 44.7% [3]. While factors such as genetics, age, gender, body mass index (BMI), and unhealthy lifestyles are known contributors to the development of hypertension, the current evidence suggests these elements only partially account for the condition’s etiology. As a result, a thorough understanding of hypertension and its associated factors may aid in the prevention and treatment of hypertension, ultimately improving the prognosis and quality of life of afflicted patients.

High-altitude polycythemia (HAPC) is a typical form of chronic mountain sickness (CMS). The key features of HAPC are compensatory hyperproliferative red blood cells and hemoglobin resulting from prolonged hypoxia. These factors increase blood viscosity, hamper microcirculation, and disrupt the immune system, which in turn induces significant organ damage and sleep-disordered breathing [4, 5]. Some investigations have observed that excessive erythrocytosis may be independently associated with hypertension [6, 7]; however, another study revealed contradictory results, showing that no association exists between them [8]. It is worth noting that there is a paucity of research into the relationship between HAPC and hypertension, specifically among populations residing at ultrahigh altitudes ranging from 3500 to 5500 m [9]. The primary aim of this study was to investigate the potential association between HAPC and hypertension among adults who reside at extreme high-altitude elevations.

## Materials and methods

### Study population

**Fig. 1:**
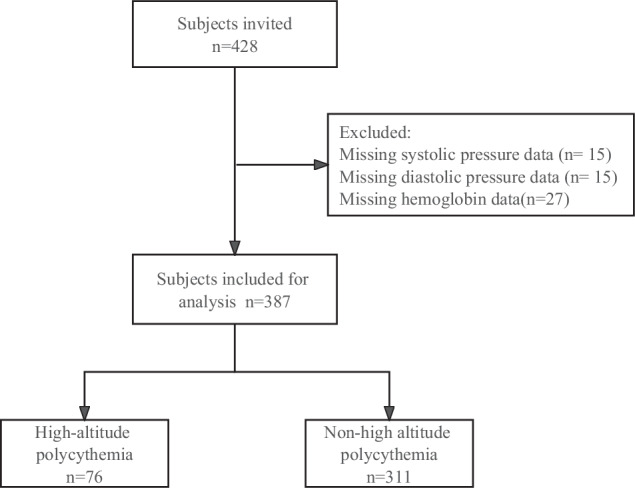
The Flow chart of the screening and enrolling of study subjects.

The Tanggula Mountains, with an average elevation of 4700 m, are situated in the Anduo County. At Anduo People’s Hospital, we conducted a cross-sectional study with 428 voluntary participants from April to May 2021. The inclusion criteria for this study were as follows: participants had to be at least 18 years, residents of the town for at least six months, and free from malignant tumors, severe kidney or liver failure, hematological system diseases, severe chronic cardiopulmonary disease, or inaccurate or missing data. Figure 1 displays the flowchart for participant enrollment. Ethical approval for conducting this study was obtained from our hospital’s ethics committee. Every subject provided informed consent. We confirmed that all methods were performed in accordance with the relevant guidelines and regulations.

### Data collection

Following the study protocol, trained research personnel conducted face-to-face interviews and physical exams on the enrolled individuals to gather relevant data. The details were as follows: Questionnaires assessed general demographic characteristics (gender, age, ethnicity, and marital status), behavioral habits (smoking and drinking status), personal and family history (history of cardiopulmonary disease, chronic kidney disease, and hematological disease), and history of taking Tibetan medicines. Physical examinations were conducted according to a standardized method of centralized measurement, encompassing assessments of blood pressure (BP), waist circumference (WC), height, weight, pulse rate (PR), and pulse oxygen saturation (SpO2). After removing their coat and shoes, each participant’s height and weight were measured with a calibrated scale. BMI was calculated as the participants’ weight in kilograms (kg) divided by the square of their height in meters (m^2^). WC was measured through the center of the umbilicus. Participants were instructed to rest for over five minutes, and the average blood pressure was determined by taking two seated measurements using an automatic sphygmomanometer. Subsequently, the pulse rate and oxygen saturation were measured on the finger after resting for 5–10 min.

### Laboratory measurements

After fasting overnight for more than 8 hours, venous blood specimens were drawn the next morning for examination within the laboratory facilities at Anduo People’s Hospital. All analyses were completed within an hour. An automatic hematology analyzer (SYSMEX XN-550) tested hematological parameters, such as hemoglobin (HGB) and hematocrit (HCT), and the following parameters were evaluated using an automatic biochemical analyzer (HITACHI-7180): total cholesterol (TC), high-density lipoprotein cholesterol (HDL-C), triglycerides (TG), serum creatinine (Scr), and fasting blood glucose (FBG).

### Definitions

Hypertension was defined as a systolic blood pressure of ≥140 mmHg or/and a diastolic blood pressure of ≥90 mmHg [10]. HAPC was defined as excessive erythrocytosis, characterized by HGB levels ≥190 g/L for females and ≥ 210 g/L for males, among individuals living above 2500 m [11]. Obesity was defined as a BMI ≥ 28 kg/m^2^, and overweight was defined as a BMI between 24 kg/m^2^ and 27.9 kg/m^2^ [12].

### Statistical analyses

Data are presented as the mean ± standard deviation (SD) or median (interquartile range) for continuous variables and as the frequency (percentage) for categorical variables. We compared the baseline characteristics of non-HAPC and HAPC groups using *t*-tests, Mann-Whitney tests, or χ² tests as appropriate. Univariate and multivariable logistic regression analyses were used to identify the association between hemoglobin and hypertension. Hemoglobin was analyzed as a categorical variable (clinical diagnosis) and as a continuous variable (per 10 g/L). Three criteria were applied to select these confounders: [1] Consideration of clinical experience and reports in the literature; [2] Inclusion of *P* values less than 0.1 in the univariate analysis; and [3] Identification of variables where the matched OR changed by over 10% after adjustment. We constructed three models: (i) Model 1 adjusted for age and gender; (ii) Model 2 adjusted for age, gender, ethnicity, BMI, and WC; and (iii) Model 3 adjusted for age, gender, ethnicity, BMI, WC, PR, Scr TC, TG, and LDL_C. To investigate the impact of various factors on the above-mentioned associations, interaction and stratified analyses were carried out with Model 3, considering gender, ethnicity, and BMI, based on the result of the univariate analysis (*P* < 0.05). All statistical analyses were performed with the software packages R 3.3.2 (http://www.Rproject.org, The R Foundation) and Free Statistics software version 1.9. A two-tailed test was performed, and *p* < 0.05 was considered statistically significant.

## Results

### Study population

Among the 428 participants initially enrolled, 48 were excluded due to missing diastolic blood pressure (*n* = 15), systolic blood pressure (*n* = 15), or hemoglobin (*n* = 27) data. Finally, 387 subjects were included in the final analysis. Figure 1 presents a flowchart of subject selection.

### Baseline characteristics in the HAPC and non-HAPC group

We included 387 participants with a mean age of 32.6 ± 6.3 years. Among them 44.7% were Hans, and males accounted for 67.2% of the total sample. Of these  participants, the overall prevalence of hypertension was 27.1%. Table 1 presents the participants’ overall features based on the presence or absence of HAPC. Compared to participants without HAPC, those with HAPC tended to be male, of Han ethnicity, and had a higher PR, TG, LDL_C, and Scr levels, along with lower SpO2 levels (all *P* < 0.05). However, the two groups did not demonstrate significant differences in body mass index, serum cholesterol, and glucose levels (all *P* > 0.05).

**Table 1:** Baseline characteristics of the study population according to non-high altitude polycythemia and high-altitude polycythemia.

Variables	Total (*n* = 387)	Non-HAPC (*n* = 311)	HAPC (*n* = 76)	*P*-value
Gender				<0.001
Male	260 (67.2)	192 (61.7)	68 (89.5)	
Female	127 (32.8)	119 (38.3)	8 (10.5)	
Age	32.6 ± 6.3	32.5 ± 6.4	32.7 ± 6.1	0.799
Marital status				0.446
Married	247 (71.4)	193 (70.4)	54 (75)	
Single	99 (28.6)	81 (29.6)	18 (25)	
Ethnicity				<0.001
Hans	173 (44.7)	121 (38.9)	52 (68.4)	
Tibetans	214 (55.3)	190 (61.1)	24 (31.6)	
Smoking				0.336
No	239 (69.5)	193 (70.7)	46 (64.8)	
Yes	105 (30.5)	80 (29.3)	25 (35.2)	
Drinking				0.207
No	216 (62.8)	176 (64.5)	40 (56.3)	
Yes	128 (37.2)	97 (35.5)	31 (43.7)	
Tibetan medicine intake				0.898
No	254 (73.8)	202 (74)	52 (73.2)	
Yes	90 (26.2)	71 (26)	19 (26.8)	
BMI (kg/m2)	24.3 ± 3.7	24.2 ± 3.8	24.6 ± 3.1	0.441
WC (cm)	84.2 ± 11.2	83.7 ± 11.3	86.4 ± 10.4	0.061
PR(bpm)	85.1 ± 12.5	84.5 ± 12.4	87.7 ± 12.8	0.044
FBG (mmol/L)	4.9 ± 1.2	5.0 ± 1.2	4.8 ± 0.9	0.252
TC (mmol/L)	4.3 ± 0.9	4.3 ± 0.8	4.5 ± 1.0	0.136
TG (mmol/L)	1.0(0.7,1,5)	1.0(0.7,1,4)	1.3(1.0,1,8)	<0.001
HDL_C(mmol/L)	0.8 ± 0.2	0.8 ± 0.1	0.8 ± 0.3	0.792
LDL_C(mmol/L)	2.8 ± 0.8	2.8 ± 0.8	3.0 ± 0.9	0.014
SpO2(%)	83.1 ± 4.2	83.5 ± 3.9	81.4 ± 4.9	<0.001
Scr(µmol/L)	89.1 ± 17.3	88.1 ± 17.5	93.4 ± 16.1	0.016

Data were mean ± SD or median (IQR) for skewed variables or numbers (proportions) for categorical variables.

*HAPC* high-altitude polycythemia, *BMI* body mass index, *WC* waist circumference, *PR* pulse rate, *SpO2* pulse oxygen saturation, *Scr* Serum creatinine, *FBG* fast blood glucose, *TC* total cholesterol, *HDL-C* high-density lipoprotein cholesterol, *LDL-C* low- density lipoprotein cholesterol, *TG* triglyceride.

### Univariate and multivariate analyses of hypertension

As shown in Table 2, in the general population, univariate analysis revealed significant associations between hypertension and BMI, WC, PR, HGB, HCT, Scr, TC, TG, and LDL_C (*P* < 0.05). Nevertheless, there was no statistically significant correlation between the FBG and hypertension. During the multivariable analyses, after adjusting for relevant variables (Table 3), hemoglobin levels expressed as a continuous variable were found to be associated with hypertension risk. Specifically, for every 10 g/L increase in hemoglobin, there was a 26% increased risk of hypertension (OR = 1.26, 95% CI; 1.11–1.44, *P* < 0.001). When hemoglobin was categorized into two groups by clinical diagnosis, a significant  risk of hypertension was found in the HAPC group compared to the non-HAPC group (OR = 3.01; 95% CI, 1.66–5.44, *P* < 0.001). All models showed consistent statistical outcomes.

**Table 2:** Results of univariate analysis of hypertension.

Variable	OR (95% CI)	*P*-value
Gender		
Male	ref	
Female	0.28 (0.15 ~ 0.5)	<0.001
Age	1 (0.97 ~ 1.04)	0.913
Ethnicity		
Hans	ref	
Tibetans	0.53 (0.34 ~ 0.83)	0.006
Marital status		
Married	ref	
Single	0.9 (0.53 ~ 1.52)	0.697
Smoking		
No	ref	
Yes	1.31 (0.79 ~ 2.16)	0.295
Drinking		
No	ref	
Yes	1.41 (0.87 ~ 2.29)	0.159
Tibetan medicine intake		
No	ref	
Yes	1.26 (0.74 ~ 2.13)	0.389
BMI (kg/m2)	1.13 (1.06 ~ 1.2)	<0.001
WC (cm)	1.05 (1.03 ~ 1.07)	<0.001
PR(bpm)	1.03 (1.01 ~ 1.05)	0.002
HGB(g/L)	1.03 (1.02 ~ 1.04)	<0.001
HCT(%)	1.11 (1.08 ~ 1.15)	<0.001
Scr(µmol/L)	1.02 (1.01 ~ 1.04)	0.001
FBG(mmol/L)	1.03 (0.84 ~ 1.28)	0.761
TC(mmol/L)	1.77 (1.33 ~ 2.34)	<0.001
TG(mmol/L)	1.83 (1.36 ~ 2.46)	<0.001
HDL_C(mmol/L)	0.2 (0.04 ~ 1.08)	<0.061
LDL_C(mmol/L)	2.06 (1.52 ~ 2.78)	<0.001
SpO2(%)	0.95 (0.9 ~ 1)	0.07

*OR* odds ratio, *CI* confidence interval, *BMI* body mass index, *WC* waist circumference, *PR* pulse rate, *HGB* hemoglobin, *Scr* Serum creatinine, *FBG* fast blood glucose, *TC* total cholesterol, *TG* triglyceride, *HDL-C* high-density lipoprotein cholesterol, *LDL-C* low-density lipoprotein cholesterol, *SpO2* pulse oxygen saturation.

**Table 3:** Multivariable logistic regression analysis of the association between hemoglobin and hypertension.

	Unadjusted		Model 1		Model 2		Model 3	
Variables	OR (95% CI)	*P*-value	OR (95% CI)	*P*-value	OR (95% CI)	*P*-value	OR (95% CI)	*P*-value
Hemoglobin per 10 g/L	1.38 (1.25 ~ 1.52)	<0.001	1.34 (1.19 ~ 1.51)	<0.001	1.3 (1.15 ~ 1.52)	<0.001	1.26 (1.11 ~ 1.44)	<0.001
Binary variables								
Non-HAPC	reference		reference		reference		reference	
HAPC	4.52 (2.67 ~ 7.67)	<0.001	3.64 (2.12 ~ 6.27)	<0.001	3.26 (1.85 ~ 5.75)	<0.001	3.01 (1.66 ~ 5.44)	<0.001

*HAPC* high-altitude polycythemia, *BMI* body mass index, *WC* waist circumference, *PR* pulse rate, *Scr* Serum creatinine, *TC* total cholesterol, *TG* triglyceride, *LDL-C* low-density lipoprotein cholesterol, *OR* odds ratio, *CI* confidence interval.

Model 1: adjusted for age, gender; Model 2: adjusted for model 1+ ethnicity +BMI + WC; Model 3: adjusted for model 2 + TC + TG + LDL_C+Scr+PR

### Subgroup analyses on the association between HAPC and hypertension

**Fig. 2:**
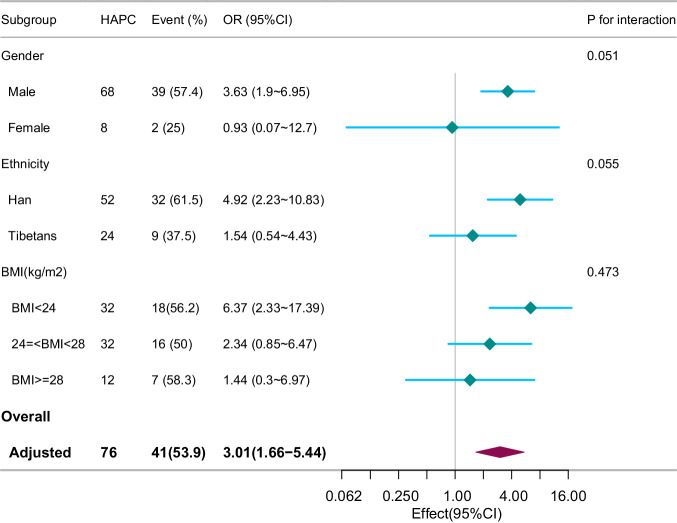
Effect size of high-altitude polycythemia on hypertension in each subgroup. Notes: OR odds ratio, CI confidence interval, BMI body mass index, WC waist circumference, PR pulse rate, Scr Serum creatinine, TC total cholesterol, TG triglyceride, LDL-C low-density lipoprotein cholesterol. adjusted for age+gender+ethnicity +BMI + WC+Scr+PR + TC + TG + LDL_C.

To determine if gender, ethnicity, or BMI altered the favorable association between high-altitude polycythemia and hypertension, stratified and interactive analyses were performed (Fig. 2). No variable exhibited an interaction effect in the association between HAPC and hypertension (P for interaction >0.05). Nevertheless, in the subgroups, male gender, Han ethnicity, and a BMI < 24 kg/m^2^ were associated with an increased risk of hypertension.

## Discussion

Through this cross-sectional investigation, we demonstrated that HAPC is independently linked to a two-fold increase in the risk of hypertension compared to the non-HAPC group. At the same time, the risk of hypertension increased by 26% for every 10 g/L rise in hemoglobin.

To the best of our knowledge, epidemiological data on the relationship between HAPC and hypertension in Tibet are limited. However, some similar studies have reported that hypertension was associated with excessive erythrocytosis (defined as hemoglobin concentration ≥210 g/L in men and 190 g/L in women) in the Andean highlanders who live in Peru [13, 14]. Despite the inclusion of Andean highlanders in both of these investigations, they largely support what we discovered. Furthermore, a recent cross-sectional study conducted in Luhuo County, Ganzi Tibetan Autonomous Prefecture (with a mean elevation of 3500 m) found that hemoglobin levels were correlated with hypertension among native Tibetans [7]. In addition to confirming this association, we offer additional information. In our study population, we confirmed the positive correlation between HAPC and hypertension in the context of adults at Tibetan ultrahigh altitudes. Aside from these widely accepted conclusions, certain findings highlighted in our study are noteworthy. First, even after adjusting for covariates, we identified a substantial positive correlation between HAPC and hypertension. Second, for the first time, we compared Tibetans and individuals of Hans in the plateau region. China has 56 ethnic groups, with the Han population predominantly settled in plains and low-altitude areas, although a minority have migrated to high-altitude regions for work or business purposes. The Tibetan people, on the other hand, have inhabited the Qinghai-Tibet Plateau for over ten thousand years [15]. Based on the subgroup analysis, we discovered that Hans individuals were more likely to have hypertension. ZhenHua Li et al. reported the same result [16]; however, they did not comprehensively explain their findings. To further understand the difference between the Tibetan and Hans populations, we compared their characteristics (Supplementary Table 1). Hans individuals had higher hemoglobin levels (197.8 ± 25.2 g/L vs. 179.6 ± 26.7 g/L), increased PRs, a higher frequency of cigarette smoking, as well as a greater risk of hypertension (34.1% vs. 21.5%), despite having a lower BMI. No significant difference in glucose levels was observed between the two groups. According to the literature, Hans Chinese individuals have a higher prevalence of HAPC [17], whereas Tibetans do not exhibit significantly elevated hemoglobin levels associated with HAPC. This difference is attributed to the genetic adaptations of Tibetans, specifically variations in the EPAS1 and EGLN1 genes, which contribute to their ability to maintain relatively low hemoglobin levels despite living at high altitudes [18]. A previous study showed that the mean blood pressure was significantly elevated in the HAPC group residing at the same elevation [19], which also supported our findings. Another intriguing finding was that similar to a Japanese study [20], significant relationships were limited to individuals who had a BMI < 24 kg/m^2^ in the subgroup analysis. As a result, further study is needed in this field to explore this phenomenon. Hence, elevated levels of hemoglobin could serve as a valuable predictor of hypertension.

Based on the findings of our investigation and previously published research, we proposed probable pathogenetic pathways that could lead to hypertension associated with HAPC. As is commonly known, people who live at high altitudes are exposed to hypobaric and hypoxic conditions for a prolonged period. Chronic hypoxia primarily drives the development of HAPC, with additional contributions from severe nocturnal hypoxemia and sleep disordered breathing in Highlanders [21, 22]. Excessive hemoglobin levels result in high blood viscosity and peripheral vascular resistance, contributing to the physiopathology of hypertension [23–25]. The concentration of hemoglobin plays a key role in vascular function. As we know, hemoglobin is highly affinitized to nitric oxide (NO) [26], a molecule crucial for regulating arterial tone. In the HAPC population, a notable release of free hemoglobin occurs, resulting in the increased scavenging of NO, which is a major cause of high blood pressure [27].

Nevertheless, it is important to take into account the limitations of the current study. First, because this is an observational study, we cannot demonstrate a causal connection between HAPC and the risk of hypertension in the Tibetan population. Second, given the modest sample size, this study was constrained in its ability to comprehensively assess statistical efficacy and investigate interactions among various factors. While preliminary findings may provide valuable insights, validation in larger cohorts would strengthen confidence in the conclusions drawn. Third, the association between HAPC and hypertension was examined after correcting for potential variables; however, it was not possible to entirely exclude the impact of additional variables not accounted for, such as proteinuria, blood uric acid, and family history of hypertension, which may have led to an overestimation of the observed associations. Nevertheless, our study population is from a single hospital and is reasonably homogeneous. Moreover, we have already accounted for several significant confounding factors in our analysis. Therefore, the absence of additional variables is unlikely to have a substantial impact on the current results. Future studies should thus aim to address these limitations to validate and expand upon our results.

## Conclusions

High-altitude polycythemia is positively associated with hypertension in adults residing at Tibetan ultrahigh altitudes. Furthermore, Hans Chinese individuals were observed to have a greater risk of developing hypertension compared to Tibetans living at these extreme elevations. These findings highlight the need for more in-depth studies and targeted healthcare initiatives focused on adults in Tibet.

## Summary

### What is known about topic:

- The association between high-altitude polycythemia and hypertension remains unclear.
- Research evidence regarding the relationship between high-altitude polycythemia and hypertension among adults living at Tibetan ultrahigh altitudes is still limited.

### What this study adds:

- This study found that high-altitude polycythemia was significantly associated with hypertension.
- The study also revealed that Hans participants, compared to Tibetan participants, had a greater risk of developing hypertension, despite living at the same high-altitude region.

### Supplementary information

supplementary table 1

## Data Availability

The authors will provide the original data unreservedly to substantiate the conclusions of this article.
