# Supplementary material for: Association between high-altitude polycythemia and hypertension: a cross-sectional study in adults at Tibetan ultrahigh altitudes
Source: J Hum Hypertens. 2024 May 27;38(7):555–60. doi: 10.1038/s41371-024-00916-3 (PMC11239486; doi:10.1038/s41371-024-00916-3)
Supplement: Supplementary file 1 — supplementary table 1 [file 41371_2024_916_MOESM1_ESM.docx]

**Supplementary table 1. Comparison of the characteristics between Hans and Tibetans in Tibet**

| Variables | Total  (n = 387) | Hans  (n = 173) | Tibetans  (n = 214) | P-value |
| --- | --- | --- | --- | --- |
| Gender | |  |  | < 0.001 |
| Male | 260 (67.2) | 132 (76.3) | 128 (59.8) |  |
| Female | 127 (32.8) | 41 (23.7) | 86 (40.2) |  |
| Age | 32.6 ± 6.3 | 30.9 ± 5.8 | 33.9 ± 6.4 | < 0.001 |
| Smoking | |  |  | 0.003 |
| No | 239 (69.5) | 93 (61.2) | 146 (76) |  |
| Yes | 105 (30.5) | 59 (38.8) | 46 (24) |  |
| Drinking | |  |  | 0.306 |
| No | 216 (62.8) | 100 (65.8) | 116 (60.4) |  |
| Yes | 128 (37.2) | 52 (34.2) | 76 (39.6) |  |
| BMI(kg/m2) | 24.3 ± 3.7 | 23.2 ± 3.3 | 25.2 ± 3.7 | < 0.001 |
| HGB(g/L) | 187.7 ± 27.6 | 197.8 ± 25.2 | 179.6 ± 26.7 | < 0.001 |
| PR(bpm) | 85.1 ± 12.5 | 86.6 ± 12.7 | 83.9 ± 12.2 | 0.037 |
| SpO2(%) | 83.1 ± 4.2 | 83.5 ± 4.4 | 82.7 ± 4.1 | 0.078 |
| Scr(µmol/L) | 89.1 ± 17.3 | 88.9 ± 16.3 | 89.3 ± 18.1 | 0.852 |
| TC(mmol/L) | 4.3 ± 0.9 | 4.3 ± 0.8 | 4.4 ± 0.9 | 0.641 |
| FBG(mmol/L) | 4.9 ± 1.2 | 5.0 ± 0.7 | 4.9 ± 1.4 | 0.094 |
| HDL_C(mmol/L) | 0.8 ± 0.2 | 0.7 ± 0.1 | 0.8 ± 0.2 | 0.002 |
| LDL_C(mmol/L) | 2.8 ± 0.8 | 2.8 ± 0.7 | 2.8 ± 0.9 | 0.881 |
| HAPC | |  |  | < 0.001 |
| No | 311 (80.4) | 121 (69.9) | 190 (88.8) |  |
| Yes | 76 (19.6) | 52 (30.1) | 24 (11.2) |  |
| Hypertension | |  |  | 0.006 |
| No | 282 (72.9) | 114 (65.9) | 168 (78.5) |  |
| Yes | 105 (27.1) | 59 (34.1) | 46 (21.5) |  |
| TG(mmol/L) | 1.0(0.7,1.5) | 1.2(0.9,1.8) | 0.9(0.7,1.3) | <0.001 |

Data were mean ± SD or median (IQR) for skewed variables or numbers (proportions) for categorical variables.

Abbreviations: HAPC, high-altitude polycythemia; BMI, body mass index; HGB, hemoglobin; PR, pulse rate, SpO2, pulse oxygen saturation; Scr, Serum creatinine; FBG, fast blood glucose; TC, total cholesterol; HDL-C, high-density lipoprotein cholesterol; LDL-C, low- density lipoprotein cholesterol; TG, triglyceride.
